# Supplementary material for: bmd: an R package for benchmark dose estimation
Source: PeerJ. 2020 Dec 17;8:e10557. doi: 10.7717/peerj.10557 (PMC7750002; doi:10.7717/peerj.10557)
Supplement: Supplemental Information 1 — Tables S1 and S2 , Figs. S1–S4, and R-code for all examples. [file peerj-08-10557-s001.docx]

Supplementary material

# Supplementary tables and figures

Table S1: Overview of different definitions of the benchmark dose available in the bmd package. An X indicates that the definition is relevant for the specific type of response data

|  |  | Available for/ relevant to | | |
| --- | --- | --- | --- | --- |
| Definition | Argument def | Binomial data | Continuous data | Count data |
| Additional | “additional” | X |  |  |
| Excess/extra | “excess” | X |  |  |
| Hybrid approach (Additional risk) | “hybridAdd” |  | X |  |
| Hybrid approach (Excess risk) | “hybridExc” |  | X |  |
| Added response | “added” |  | X | X |
| Relative response | “relative” |  | X | X |
| Extra response | “extra” |  | X | X |
| Directly defined | “point” | X | X | X |

Table S2: Overview of different models available for benchmark dose estimation using the bmd package. Most of the models are directly available in drc or bmd. Some are achieved by fixing a parameter.

| Model name | Number of parameters | Model function |
| --- | --- | --- |
| Log-logistic/  Hill model | 4 | $f\left( x,\left( b,c,d,e \right) \right)=c+(d-c){(1+{(x/e)}^{b} )}^{-1}$ |
| Log-logistic | 3 | $f\left( x,\left( b,d,e \right) \right)=d{(1+{(x/e)}^{b} )}^{-1}$ |
|  | 2 | $f\left( x,\left( b,e \right) \right)={(1+{(x/e)}^{b} )}^{-1}$ |
| Shifted E-max | 3 | $f\left( x,\left( c,d,e \right) \right)=c+(d-c)x{(x+e )}^{-1}$ |
| E-max/  Michaelis-Menten | 2 | $f\left( x,\left( d,e \right) \right)=dx{(x+e )}^{-1}$ |
| Burr type III/  Log-logistic | 5 | $f\left( x,\left( b,c,d,e,f \right) \right)=c+(d-c){(1+{(x/e)}^{b} )}^{-f}$ |
| Log-normal | 4 | $f\left( x,\left( b,c,d,e \right) \right)=c+(d-c)\phi(b\left( \log\left( x \right)-\log\left( e \right) \right))$ |
|  | 3 | $f\left( x,\left( b,d,e \right) \right)=d\phi(b\left( \log\left( x \right)-\log\left( e \right) \right))$ |
|  | 2 | $f\left( x,\left( b,e \right) \right)=\phi(b\left( \log\left( x \right)-\log\left( e \right) \right))$ |
| Weibull type 1 | 4 | $f\left( x,\left( b,c,d,e \right) \right)=c+(d-c)\exp(-{(x/e)}^{b} )$ |
|  | 3 | $f\left( x,\left( b,d,e \right) \right)=d\exp(-{(x/e)}^{b} )$ |
|  | 2 | $f\left( x,\left( b,e \right) \right)=\exp(-{(x/e)}^{b} )$ |
| Exponential decay | 3 | $f\left( x,\left( c,d,e \right) \right)=c+(d-c)exp(-x/e)$ |
|  | 2 | $f\left( x,\left( d,e \right) \right)=dexp(-x/e)$ |
| Weibull type 2 | 4 | $f\left( x,\left( b,c,d,e \right) \right)=c+(d-c)(1-\exp(-{(x/e)}^{b} ))$ |
|  | 3 | $f\left( x,\left( b,d,e \right) \right)=d(1-\exp(-{(x/e)}^{b} ))$ |
| Weibull type 2/ Extreme value model | 2 | $f\left( x,\left( b,e \right) \right)=1-\exp(-{(x/e)}^{b} )$ |
| Asymptotic regression/ First-order multistage model | 3 | $f\left( x,\left( c,d,e \right) \right)=c+(d-c)(1-\exp(-x/e ))$ |
| Second-order multistage model | 4 | $f\left( x,\left( b,c,d,e \right) \right)=c+(d-c)\exp(-b_{1}x-b_{2}x^{2} ))$ |
| No effect concentration (NEC) | 4 | $f\left( x,\left( b,c,d,e \right) \right)=\left\{ \begin{matrix} c+(d-c)exp(b(x-e)) & \mathrm{if} x>e \\ d & \mathrm{if} x\leq e \end{matrix} \right.$ |
| Fractional polynomial | 4 | $f\left( x,\left( b,c,d,e \right) \right)=$  $c+(d-c){(1+{exp(b(log(x+1))}^{p_{1}}+{e\left( \log\left( x+1 \right) \right)}^{p_{2}} ))}^{-1}$ |
| Isotonic regression model |  | $f\left( x \right)=\left\{ \begin{matrix} \tilde{y}_{i} & \mathrm{if} x=x_{i} \\ \tilde{y}_{i}+(\tilde{y}_{i+1}-\tilde{y}_{i})/(x_{i+1}-x_{i})(x-x_{i}) & \mathrm{if} x\leq e \end{matrix} \right.$,  where $\tilde{y_{i}}$ is a monotonized sequence of responses |


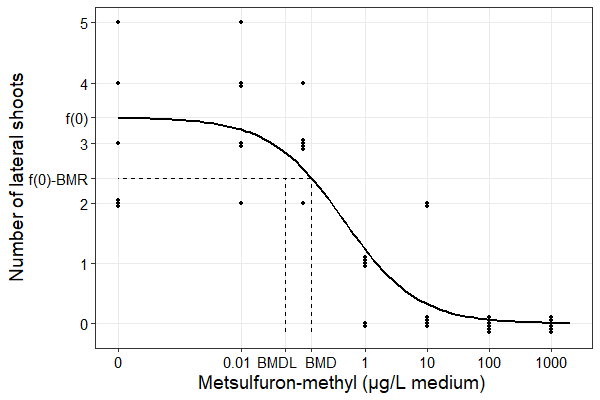


Figure S1: : The fitted concentration-response curve for the three-parameter log-logistic model fitted to data from a toxicity test with metsulfuron-methyl (µg/L medium) on the aquatic plant Elodea canadensis (Cedergreen, Streibig & Spliid, 2004). The curve is shown together with all data points, and the estimated benchmark dose (BMD) and benchmark dose lower limit (BMDL) for the added definition and a benchmark response (BMR) of 1. f(0) is the model-based estimated number of offspring for the background population. Data points close are identical in value but noise has been added to make them all visible.


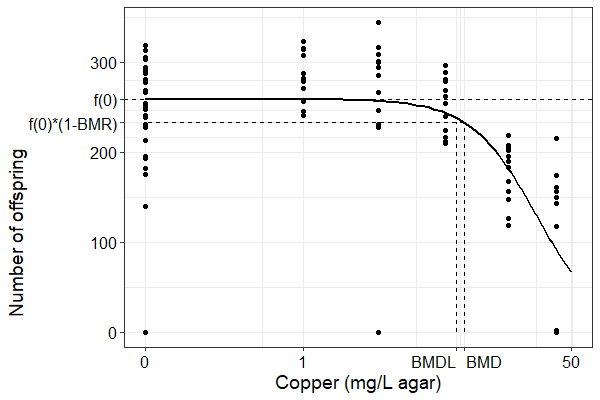


Figure S2: Fitted concentration-response curve for the three-parameter log-logistic model fitted to data from the toxicity test with copper (mg/L agar) under varying temperature (Cedergreen et al., 2016). The curve is shown together with all data points, and the estimated benchmark dose (BMD) and benchmark dose lower limit (BMDL) for the relative definition and the benchmark response (BMR) of 0.1. f(0) is the model-based estimated number of offspring for the background population.


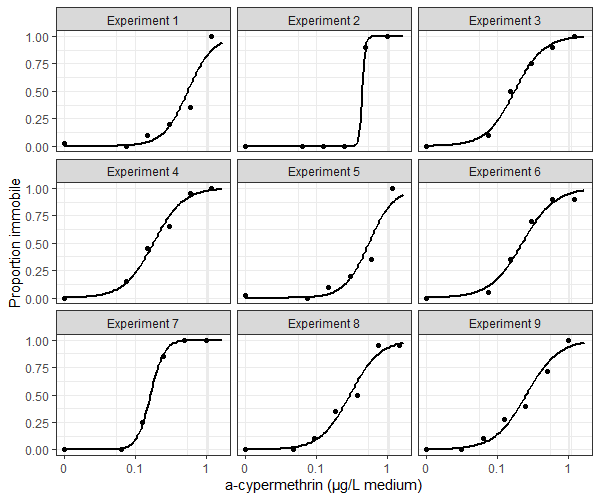


Figure S3: Fitted concentration-response curves based on a two-parameter log-logistic model for each of the nine sub-experiments in the α-cypermethrin data (Gottardi & Cedergreen, 2019).


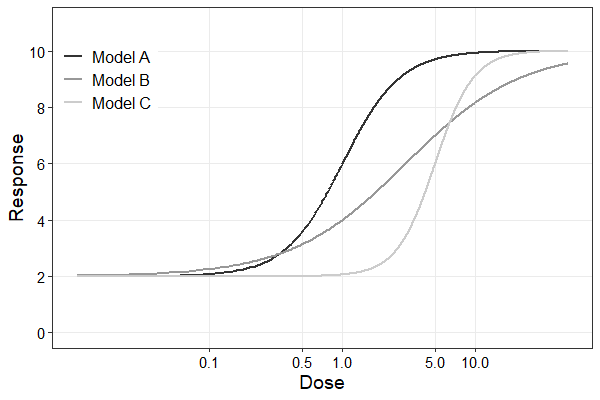


Figure S4: Three models used for simulating data for validating the **R** package bmd.

# R code for the examples

## Loading packages

The R-package *bmd* is located on GitHub in the repository doseResponse together with the latest version of *drc*.

library(devtools)
install_github("DoseResponse/drc")
install_github("DoseResponse/drcData")
install_github("DoseResponse/bmd")
library(drc)
library(bmd)
library(drcData)
library(ggplot2)
library(plyr)

## Example 3.1. Binomial data: An earthworm toxicity test with chloroacetamide

Data is found in the R-package *drcData*, with the name “chlorac”.

head(chlorac)

## conc total num.dead
## 1 0 40 3
## 2 10 40 5
## 3 20 40 6
## 4 40 40 38
## 5 80 40 40
## 6 160 40 40

Fitting a 3-parameter log-logistic model with upper limit fixed at 1 using drm() in *drc*

chlorac.LN.3 <- drm(num.dead/total ~ conc, weights = total, data = chlorac, fct = LN.3u(), type = "binomial")

Plotting the fitted model. For a nicer plot using *ggplot2* we refer to Ritz et al. 2015.

plot(chlorac.LN.3, broken = TRUE, ylim = c(0,1))


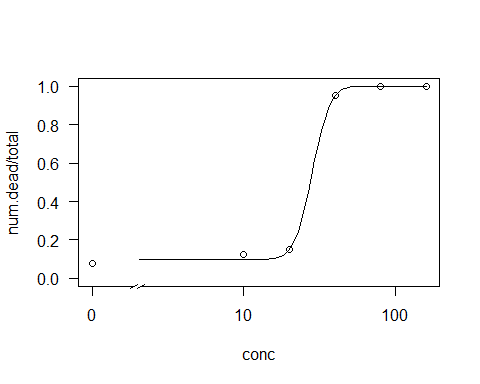


Summary of the fitted model

summary(chlorac.LN.3)

##
## Model fitted: Log-normal with upper limit at 1 (3 parms)
##
## Parameter estimates:
##
## Estimate Std. Error t-value p-value
## b:(Intercept) 4.603773 1.043813 4.4105 1.031e-05 ***
## c:(Intercept) 0.099988 0.033573 2.9783 0.002899 **
## e:(Intercept) 28.291922 2.271962 12.4526 < 2.2e-16 ***
## ---
## Signif. codes: 0 '***' 0.001 '**' 0.01 '*' 0.05 '.' 0.1 ' ' 1

Estimating the BMD and BMDL for BMR=0.05 using the excess risk definition

bmd(chlorac.LN.3, bmr=0.05, backgType = "modelBased", def="excess")

## BMD BMDL
## 19.79229 15.15075

Estimating the BMD and BMDL for BMR=0.05 using the additional risk definition

bmd(chlorac.LN.3, bmr = 0.05, backgType = "modelBased", def = "additional")

## BMD BMDL
## 20.0155 15.39552

## Example 3.2. Count data: A toxicity test in aquatic plants

Data are provided in the csv-file Elodea provided as supplementary data

Elodea <- read.csv2(file.choose())

Fitting a 3-parameter log-logistic model with a lower limit of 0 and assuming a Poisson distribution using drm() in *drc*.

Elodea.m1 <- drm(LateralCount ~ Conc,
 data = Elodea,
 type = "Poisson",
 fct = LL.3())

Plotting the fitted model.

plot(Elodea.m1, broken = TRUE, type = "all")


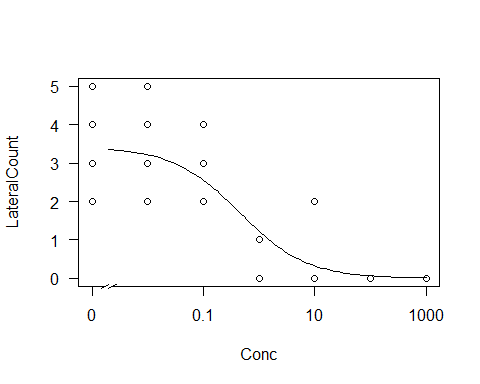


Making a residual plot

plot(resid(Elodea.m1) ~ predict(Elodea.m1))


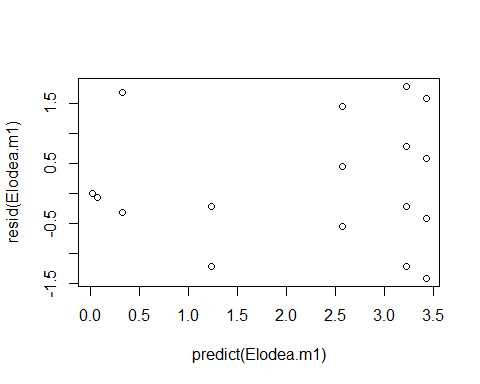


Estimating the BMD and BMDL for BMR=1 using the added definition for the delta method, inverse regression and bootstrap

bmdBoot(Elodea.m1, bmr = 1, def = "added", backgType = "modelBased")

## BMD BMDL
## 0.1331006 0.05251343

Fitting a 3-parameter log-logistic model with a lower limit of 0 and assuming a Normal distribution.

Elodea.m2 <- drm(LateralCount ~ Conc,
 data = Elodea,
 fct = LL.3())

Estimating the BMD and BMDL based on the new model for BMR=1 using the added definition for the delta method, inverse regression and bootstrap

bmdBoot(Elodea.m2, bmr=1, def="added", backgType = "modelBased")

## BMD BMDL
## 0.2543411 0.09497352

##

## Example 3.3. Count data: A toxicity test with copper under varying temperature - Count data

Data are provided in the csv-file VarTemp provided as supplementary data

CopperTemp <- read.csv2(file.choose())

Fitting a 3-parameter log-logistic model with a lower limit of 0 and assuming a Poisson distribution using drm() in *drc*.

CopperTemp.m1 <- drm(Offspring ~ Conc,
 data = CopperTemp,
 type = "Poisson",
 fct = LL.3())

Plotting the fitted model. Again, for a nicer plot using *ggplot2* we refer to Ritz et a. 2015.

plot(CopperTemp.m1, broken=TRUE, type="all")


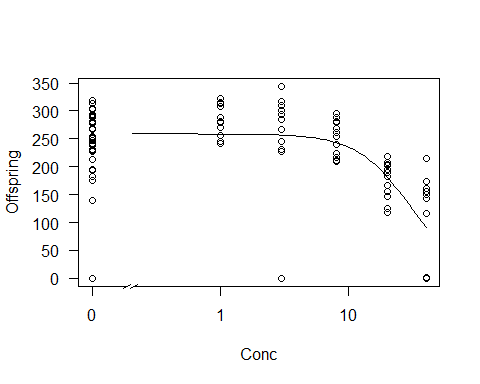


Estimating the BMD and BMDL for BMR=0.1 using the relative definition

bmd(CopperTemp.m1,
 bmr = 0.1,
 backgType = "modelBased",
 def = "relative")

## BMD BMDL
## 10.47006 9.403057

Fitting a new model where the number of offsprings are weighted by the number of days the worm was alive

CopperTemp.m2 <- drm(Offspring ~ Conc,
 data = CopperTemp,
 weights = Lifespan,
 type = "Poisson",
 fct = LL.3())

Estimating BMD and BMDL for this new model, still having BMR=0.1 and using the relative definition

bmd(CopperTemp.m2,
 bmr = 0.1,
 backgType = "modelBased",
 def = "relative")

## BMD BMDL
## 31.16192 28.89062

Taking overdispersion into account when estimating BMD and BMDL using the sandwich variance estimates

library(sandwich)

bmd(CopperTemp.m2,
 bmr = 0.1,
 backgType = "modelBased",
 def = "relative",
 sandwich.vcov = TRUE)

## BMD BMDL
## 31.16192 18.62904

Fitting the model again, this time assuming data follow a negative binomial distribution. This is another way to account for overdispersion

CopperTemp.m3 <- drm(Offspring ~ Conc,
 data = CopperTemp,
 weights = Lifespan,
 type = "negbin2",
 fct = LL.3())

Estimating BMD and BMDL for the model assuming a negative binomial distribution

bmd(CopperTemp.m3,
 bmr = 0.1,
 backgType = "modelBased",
 def = "relative")

## BMD BMDL
## 27.98813 15.99371

##

## Example 3.4 Continuous data: A fish test

Data can be found in *drcData* with the name o.mykiss.

We start by removing the missing values

O.mykiss.c <- na.omit(O.mykiss)

Fitting a 2-parameter exponential decay model to data using drm() in *drc*

O.mykiss.EXD.2 <- drm(weight ~ conc,
 data = O.mykiss.c,
 fct = EXD.2())

Plotting the fitted model

plot(O.mykiss.EXD.2, broken = TRUE, type = "all",
 xlim = c(0, 500), ylim = c(0, 5),
 xlab = "Concentration (mg/L)", ylab = "Wet weight (g)")


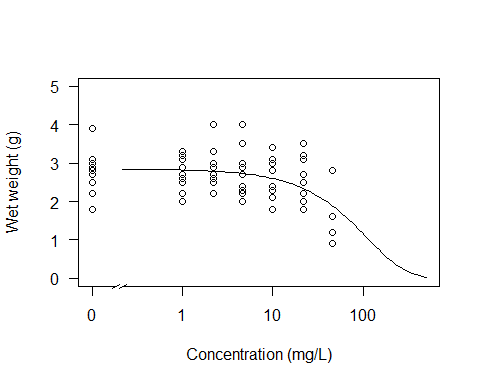


Residual plot, checking for model fit and variance homogeneity

plot(resid(O.mykiss.EXD.2)~fitted(O.mykiss.EXD.2))


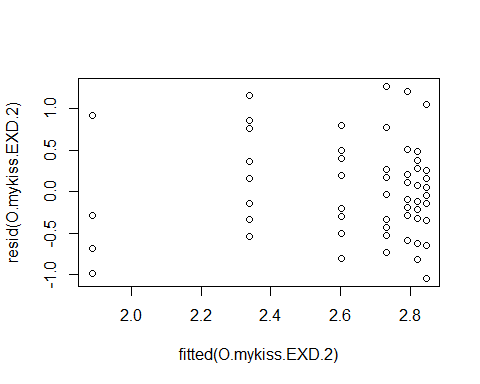


QQ-plot, checking for normality

qqnorm(resid(O.mykiss.EXD.2))
qqline(resid(O.mykiss.EXD.2))


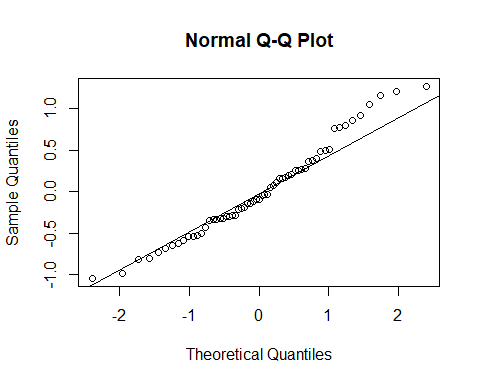


A BMD associated with a BMR=0.05 using the hybrid approach with 2 SD as the cutoff is estimated as follows.

bmd(O.mykiss.EXD.2,
 bmr = 0.05,
 backgType = "hybridSD",
 def = "hybridAdd",
 backg = 2)

## BMD BMDL
## 12.65354 6.371416

Fitting a 3-parameter log-logistic model, and two different 3-parameter Weibull models to data

O.mykiss.LL.3 <- drm(weight ~ conc,
 data = O.mykiss,
 fct = LL.3(),
 na.action = na.omit)

O.mykiss.W1.3 <- drm(weight ~ conc,
 data = O.mykiss,
 fct = W1.3(),
 na.action = na.omit)

O.mykiss.W2.3 <- drm(weight ~ conc,
 data = O.mykiss,
 fct = W2.3(),
 na.action = na.omit)

Comparing model fits with AIC

AIC(O.mykiss.EXD.2, O.mykiss.LL.3, O.mykiss.W1.3, O.mykiss.W2.3)

## df AIC
## O.mykiss.EXD.2 3 106.3066
## O.mykiss.LL.3 4 106.6501
## O.mykiss.W1.3 4 106.5823
## O.mykiss.W2.3 4 106.9406

Estimating BMD and BMDL for the 3 additional models for BMR=0.05 using the hybrid approach with 2 SD as the cutoff

bmd(O.mykiss.LL.3,
 bmr = 0.05,
 backgType = "hybridSD",
 def = "hybridAdd",
 backg = 2)

## BMD BMDL
## 22.91039 9.046736

bmd(O.mykiss.W1.3,
 bmr = 0.05,
 backgType = "hybridSD",
 def = "hybridAdd",
 backg = 2)

## BMD BMDL
## 22.71909 8.124225

bmd(O.mykiss.W2.3,
 bmr = 0.05,
 backgType = "hybridSD",
 def = "hybridAdd",
 backg = 2)

## BMD BMDL
## 24.25913 14.2518

Plotting all models in the same figure

plot(O.mykiss.EXD.2, broken = TRUE, type = "all",
 xlim = c(0, 500), ylim = c(0, 5),
 xlab = "Concentration (mg/L)", ylab = "Wet weight (g)")
plot(O.mykiss.LL.3, broken = TRUE, type = "all",
 xlim = c(0, 500), ylim = c(0, 5),
 add = TRUE, lty = 2)
plot(O.mykiss.W1.3, broken = TRUE, type = "all",
 xlim = c(0, 500), ylim = c(0, 5),
 add = TRUE, lty = 3)
plot(O.mykiss.W2.3, broken = TRUE, type = "all",
 xlim = c(0, 500), ylim = c(0, 5),
 add = TRUE, lty = 4)


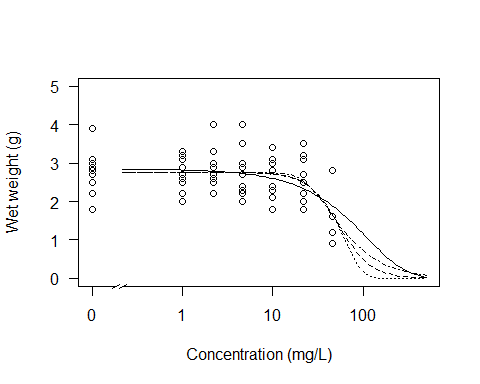


Estimating BMD and BMDL based on model averaging using the Buckland definition for estimating BMDL

modelList<-list(O.mykiss.EXD.2,O.mykiss.LL.3,O.mykiss.W1.3,O.mykiss.W2.3)
bmdMA(modelList, modelWeights="AIC",
 bmr=0.05,
 backgType = "hybridSD",
 def="hybridAdd",
 backg = 2,
 type="Buckland")

## BMD_MA BMDL_MA
## 19.68024 5.804238

Estimating BMD and BMDL based on model averaging using bootstrap for estimating BMDL. The output will give an error message for each bootstrap sample with a non-converging model. These models are disregarded in the bootstrap results, and can therefore be ignored.

modelList<-list(O.mykiss.EXD.2,O.mykiss.LL.3,O.mykiss.W1.3,O.mykiss.W2.3)
bmdMA(modelList, modelWeights="AIC",
 bmr=0.05,
 backgType = "hybridSD",
 def="hybridAdd",
 backg = 2,
 type="bootstrap")

## Error in optim(startVec, opfct, hessian = TRUE, method = optMethod, control = list(maxit = maxIt, :
## Error in optim(startVec, opfct, hessian = TRUE, method = optMethod, control = list(maxit = maxIt, :

## non-finite finite-difference value [1]
## Error in optim(startVec, opfct, hessian = TRUE, method = optMethod, control = list(maxit = maxIt, :
## non-finite finite-difference value [2]
## Error in optim(startVec, opfct, hessian = TRUE, method = optMethod, control = list(maxit = maxIt, :
## non-finite finite-difference value [3]
## Error in optim(startVec, opfct, hessian = TRUE, method = optMethod, control = list(maxit = maxIt, :
## non-finite finite-difference value [3]
## Error in optim(startVec, opfct, hessian = TRUE, method = optMethod, control = list(maxit = maxIt, :

## BMD_MA BMDL_MA
## 19.68024 8.604666

Estimating BMD and BMDL based on model averaging of entire curves using bootstrap for estimating BMDL.

bmdMA(modelList, modelWeights="AIC",
 bmr=0.05,
 backgType = "hybridSD",
 def="hybridAdd",
 backg = 2,
 type="curve")

## Error in optim(startVec, opfct, hessian = TRUE, method = optMethod, control = list(maxit = maxIt, :
## non-finite finite-difference value [2]
## Error in optim(startVec, opfct, hessian = TRUE, method = optMethod, control = list(maxit = maxIt, :
## non-finite finite-difference value [2]
## Error in optim(startVec, opfct, hessian = TRUE, method = optMethod, control = list(maxit = maxIt, :
## non-finite finite-difference value [2]
## Error in optim(startVec, opfct, hessian = TRUE, method = optMethod, control = list(maxit = maxIt, :
## non-finite finite-difference value [2]
## non-finite finite-difference value [2]
## Error in optim(startVec, opfct, hessian = TRUE, method = optMethod, control = list(maxit = maxIt, :
## non-finite finite-difference value [3]
## Error in optim(startVec, opfct, hessian = TRUE, method = optMethod, control = list(maxit = maxIt, :
## non-finite finite-difference value [3]
## Error in optim(startVec, opfct, hessian = TRUE, method = optMethod, control = list(maxit = maxIt, :
## non-finite finite-difference value [3]
## Error in optim(startVec, opfct, hessian = TRUE, method = optMethod, control = list(maxit = maxIt, :
## non-finite finite-difference value [3]
## Error in optim(startVec, opfct, hessian = TRUE, method = optMethod, control = list(maxit = maxIt, :
## non-finite finite-difference value [3]

## BMD_MA BMDL_MA
## 20.354 8.202808

##

## Example 3.5. Binomial data in a hierarchical design: An acute toxicity test with alpha-cypermethrin

Data may be found in the data set alpha_cyp.csv provided as supplementary data

alpha.cyp <- read.csv2(file.choose())

Forming a new variable indicating the number of immobile daphnia

alpha.cyp[["Immobile"]] <- with(alpha.cyp, Total-Mobile)

Fititing a 2-parameter log-logistic model for each subset of data defined by each sub-experiment using *drc*

alpha.m1<-drm(Immobile/Total ~ Dose, weights = Total,
 data = subset(alpha.cyp, Exp == "Exp1"), type="binomial", fct = LL.2())
alpha.m2<-drm(Immobile/Total ~ Dose, weights = Total,
 data = subset(alpha.cyp, Exp == "Exp2"), type="binomial", fct = LL.2())
alpha.m3<-drm(Immobile/Total ~ Dose, weights = Total,
 data = subset(alpha.cyp, Exp == "Exp3"), type="binomial", fct = LL.2())
alpha.m4<-drm(Immobile/Total ~ Dose, weights = Total,
 data = subset(alpha.cyp, Exp == "Exp4"), type="binomial", fct = LL.2())
alpha.m5<-drm(Immobile/Total ~ Dose, weights = Total,
 data = subset(alpha.cyp, Exp == "Exp5"), type="binomial", fct = LL.2())
alpha.m6<-drm(Immobile/Total ~ Dose, weights = Total,
 data = subset(alpha.cyp, Exp == "Exp6"), type="binomial", fct = LL.2())
alpha.m7<-drm(Immobile/Total ~ Dose, weights = Total,
 data = subset(alpha.cyp, Exp == "Exp7"), type="binomial", fct = LL.2())
alpha.m8<-drm(Immobile/Total ~ Dose, weights = Total,
 data = subset(alpha.cyp, Exp == "Exp8"), type="binomial", fct = LL.2())
alpha.m9<-drm(Immobile/Total ~ Dose, weights = Total,
 data = subset(alpha.cyp, Exp == "Exp9"), type="binomial", fct = LL.2())

Estimating BMD and BMDL for each subset of data defnined by each sub-experiment using BMR=0.05 and the excess definition

alpha.m1.bmd <-bmd(alpha.m1, bmr = 0.05, backgType = "modelBased", def = "excess")

## BMD BMDL
## 0.1696149 0.1004246

alpha.m2.bmd <-bmd(alpha.m2, bmr = 0.05, backgType = "modelBased", def = "excess")

## BMD BMDL
## 0.386818 -0.5760919

alpha.m3.bmd <-bmd(alpha.m3, bmr = 0.05, backgType = "modelBased", def = "excess")

## BMD BMDL
## 0.04421228 0.02135657

alpha.m4.bmd <-bmd(alpha.m4, bmr = 0.05, backgType = "modelBased", def = "excess")

## BMD BMDL
## 0.04364098 0.0204931

alpha.m5.bmd <-bmd(alpha.m5, bmr = 0.05, backgType = "modelBased", def = "excess")

## BMD BMDL
## 0.1696149 0.1004246

alpha.m6.bmd <-bmd(alpha.m6, bmr = 0.05, backgType = "modelBased", def = "excess")

## BMD BMDL
## 0.04767556 0.02138498

alpha.m7.bmd <-bmd(alpha.m7, bmr = 0.05, backgType = "modelBased", def = "excess")

## BMD BMDL
## 0.08687902 0.0640227

alpha.m8.bmd <-bmd(alpha.m8, bmr = 0.05, backgType = "modelBased", def = "excess")

## BMD BMDL
## 0.07183345 0.03979943

alpha.m9.bmd <-bmd(alpha.m9, bmr = 0.05, backgType = "modelBased", def = "excess")

## BMD BMDL
## 0.05533272 0.02903962

As an alternative to fitting each model and obtaining BMD and BMDL for each model seperately the following approach can be used:

Define a function that fits a 2-paramter log-logistic model to a data set

fitFct.LL.2 <- function(dataSet){
drm(Immobile/Total ~ Dose, weights = Total, data = dataSet, type = "binomial", fct= LL.2())
}

Run the function on each subset of data defined by experiment (Exp)

library(plyr)
modelFits <- dlply(alpha.cyp, .(Exp), fitFct.LL.2)

For each element of the resulting list, estimate BMD and BMDL (using lapply())

BMDList <- lapply(modelFits,
 FUN = function(x){
 bmd(x, bmr = 0.05, backgType = "modelBased", def = "excess", display = FALSE)
 }
 )

Extract all BMD and BMDLs from the resulting list

BMDVec <- sapply(BMDList, FUN = function(x) {x$Results[1]})
SEVec <- sapply(BMDList, FUN = function(x) {x$SE})

Make a data set containing information about BMD, standard error for BMD, and any details related to the design. Here the data set is formed based on the vecters found above, but it could also be made “by hand”, based on the results found for each individual model fit, i.e. from alpha.m1.bmd, alpha.m2.bmd etc..

step2Data<-data.frame(BMD = BMDVec,
 SE = SEVec,
 Exp = names(BMDVec))

The resulting data set should like something like this:

head(step2Data)

## BMD SE Exp
## Exp1 0.16961488 0.04206472 Exp1
## Exp2 0.38681802 0.58540765 Exp2
## Exp3 0.04421228 0.01389528 Exp3
## Exp4 0.04364098 0.01407291 Exp4
## Exp5 0.16961488 0.04206472 Exp5
## Exp6 0.04767556 0.01598354 Exp6

Fitting a meta-analytic model to data using the package *metafor*

library(metafor)

## Loading required package: Matrix

## Loading 'metafor' package (version 2.4-0). For an overview
## and introduction to the package please type: help(metafor).

meta.m1 <- rma(BMD,
 SE^2,
 data = step2Data,
 level = 0.9)

Summary of the model fit

summary(meta.m1)

##
## Random-Effects Model (k = 9; tau^2 estimator: REML)
##
## logLik deviance AIC BIC AICc
## 11.2866 -22.5733 -18.5733 -18.4144 -16.1733
##
## tau^2 (estimated amount of total heterogeneity): 0.0007 (SE = 0.0006)
## tau (square root of estimated tau^2 value): 0.0260
## I^2 (total heterogeneity / total variability): 65.62%
## H^2 (total variability / sampling variability): 2.91
##
## Test for Heterogeneity:
## Q(df = 8) = 21.1721, p-val = 0.0067
##
## Model Results:
##
## estimate se zval pval ci.lb ci.ub
## 0.0705 0.0116 6.0573 <.0001 0.0514 0.0897 ***
##
## ---
## Signif. codes: 0 '***' 0.001 '**' 0.01 '*' 0.05 '.' 0.1 ' ' 1
